# Practical Notes and Formulæ

**Published:** 1885-12-20

**Authors:** 


### PRACTICAL NOTES AND FORMULÆ.

**Bromidia.**—A correspondent of the Druggists Circular and Chemical Gazette furnishes the following formula for "Bromidia" as used in the West Virginia Asylum for the Insane :

℞. Potas. brom., Chloral hydrate, } aa.........................ʒ ij,

Ext. cannabis ind., Ext. hyoscyamus, } aa......................gr. j,

Alcohol.....................................fl ʒ j,

Aquæ, q. s. ft.............................fl. ℥ j. M.

Et filter.

C. Wilcox substitutes simple syrup for the alcohol and water.

Another correspondent offers the following, which he has used for some time with good results :

℞. Brom. pot., Chloral hydrate, } aa.........................℥ iv,

Sol. ext. cannabis indica, Sol. ext. hyoscyamus, } aa..................gr. xvi,

Alcohol.....................................ʒ ij,

Soft water, q. s. ad..........................Oj. M.

Rub extracts in glass mortar with alcohol until dissolved ; rub salts to a powder, and mix. Then add hot water, triturate until dissolved, then filter. Care must be taken that soft water is used.

**Therapeutics of Cephalalgia.**—Prof. A. A. Smith, of New York, communicates as follows to the Medical World :

*In Nervous Headache.*—Bromide of sodium, combined with valerian, is of great service :

℞. Elix. valerian ammon.........................℥ ii,
Sodii bromidi.................................ʒ iv. M.

Sig. One teaspoonful in a wineglass of water until relieved.

*In Anæmic Headache*, ʒ ss. doses of sal-volatile, or alcohol judiciously used, are useful ; and, temporarily, a little cotton dipped in nitrate of amyl, and placed in the nostril· Cod-liver oil and iron to relieve the cause.

*In Sick Headache*, emetics, when due to gastric disturbances. When due to derangements of the nervous system, citrate of caffeine, gr. i, dose every half-hour for an hour and a half, to be repeated after three hours, if necessary.

*In Rheumatic Headache*, salicylate of soda, gr. v doses, every half hour, in aq. menth. pip.

*In Neuralgic Headache*, *m* iii of fluid extract of gelsemium, given every half-hour until the eyelids are affected, is of much service. When of malarial origin, quinine should be added to the treatment, or liq. potass. arsenitis, *m* v, and tinct. belladonnæ, *m* v, in a suitable menstruum, three times a day.

*In Dyspeptic Headache*, either mineral acids or alkalies are indicated, or salicylic acid and bromide of ammonium, according to whether the trouble be of hepatic, gastric or intestinal origin.

"*Alcoholic*" *Headaches* should be treated by a cathartic, followed by spt. ammon., arom., tinct. capsici, and spt. camph.; and taking care to secure sleep and good nourishing food.

*Gouty Headache* calls for Carisbad salts and alkalies. The following mixture is useful:

℞. Vin. colchici sem........................ ʒ iss,
Fl. ext. taraxaci........................ ℥ ii. . M.
Sig. One drachm three times a day until laxative results.

Professor Da Costa advises the application of a 4-per cent. solution of cocaine to the mucous membrane of the nose in rose cold, hay fever, and influenza.—*College and Clinical Record.*

Professor Parvin (College and Clinical Record) uses, as a local application in eczema of the vulva, the following:

℞. Plumbi acet........................ ʒ j,
Acid. hydrocyan. dil........................ f. ʒ ij,
Aquæ........................ f. ℥ iij. M.
Sig. Apply to parts every three or four hours; less often if the skin is broken.

Professor Thomson directed (College and Clinical Record), in a severe case of purulent ophthalma in an infant, that the eyes be well washed out every two or three hours with a solution of borax, 10 grains to the ounce; then apply one of these collyria:

℞. Aluminis........................ gr. ij,
Aquæ........................ f. ℥ j. M.
Or—
℞. Argent. nitratis........................ gr. ij,
Aquæ........................ f. ℥ j. M.

In dysmenorrhœa, when neuralgic in character (College and Clinical Record), Professor Bartholomew strongly advises the use of—

℞. Ext. belladonnæ........................ gr. iv,

Ext. hyoscyami, } aa........................ gr. v,

Ext. stramonii, }

Quininæ sulphat........................ ℈ ij. M.

Ft. pil. No. xx.

Sig. One pill ter die.

For amenorrhœa dependent upon debility, Professor Parvin advises (Medical World) the following, speaking very highly of its utility:

℞. Ferri sulphat. exsic........................ gr. j,

Aloes........................ gr. j,

Olei terebinthinæ........................ *m*. j. M.

Ft. pil. Sig. One ter die.

